# Competition and facilitation structure plant communities under nurse tree canopies in extremely stressful environments

**DOI:** 10.1002/ece3.2690

**Published:** 2017-03-21

**Authors:** Ali A. Al‐Namazi, Magdy I. El‐Bana, Stephen P. Bonser

**Affiliations:** ^1^ Evolution and Ecology Research CentreSchool of Biological, Earth and Environmental SciencesUNSW AustraliaSydneyNSWAustralia; ^2^ King Abdulaziz City for Science and Technology (KACST)RiyadhSaudi Arabia; ^3^ Department of BotanyFaculty of SciencePort Said UniversityPort SaidEgypt

**Keywords:** arid environments, competition, facilitation, species distributions, stress gradient hypothesis, stress tolerance

## Abstract

Nurse plant facilitation in stressful environments can produce an environment with relatively low stress under its canopy. These nurse plants may produce the conditions promoting intense competition between coexisting species under the canopy, and canopies may establish stress gradients, where stress increases toward the edge of the canopy. Competition and facilitation on these stress gradients may control species distributions in the communities under canopies. We tested the following predictions: (1) interactions between understory species shift from competition to facilitation in habitats experiencing increasing stress from the center to the edge of canopy of a nurse plant, and (2) species distributions in understory communities are controlled by competitive interactions at the center of canopy, and facilitation at the edge of the canopy. We tested these predictions using a neighbor removal experiment under nurse trees growing in arid environments. Established individuals of each of four of the most common herbaceous species in the understory were used in the experiment. Two species were more frequent in the center of the canopy, and two species were more frequent at the edge of the canopy. Established individuals of each species were subjected to neighbor removal or control treatments in both canopy center and edge habitats. We found a shift from competitive to facilitative interactions from the center to the edge of the canopy. The shift in the effect of neighbors on the target species can help to explain species distributions in these canopies. Canopy‐dominant species only perform well in the presence of neighbors in the edge microhabitat. Competition from canopy‐dominant species can also limit the performance of edge‐dominant species in the canopy microhabitat. The shift from competition to facilitation under nurse plant canopies can structure the understory communities in extremely stressful environments.

## 1 Introduction

The interactions between plants play a vital role in structuring communities (e.g., Callaway & Walker, 1997; Hacker & Bertness, 1999). Competition for limited resources typically reduces the growth rate and fitness of affected individuals (e.g., Gurevitch, Morrison, & Hedges, 2000). More broadly, competition can structure communities and control species distributions as strong competitors can exclude weak competitors from a community (Connell, 1983; Fowler, 1986). Interactions between individuals can also be positive (i.e., facilitation) through plants enhancing the establishment, growth, and survival of other plants (Callaway, 2007), and improving the microclimatic conditions and soil physical and chemical properties (Bonanomi, Incerti, & Mazzoleni, 2011; Brooker et al., 2008; Maestre et al., 2010). The interplay between positive and negative interactions between plants should be important in understanding species distributions, community structure, and ecosystem function (Goldberg, Rajaniemi, & Stewart‐Oaten, 1999; Kikvidze et al., 2011).

The prediction that interactions should shift from negative to positive across gradients of increasing stress has been formalized as the stress gradient hypothesis—SGH (Bertness & Callaway, 1994). There are studies supporting the predictions of SGH (Brooker, 2006; Callaway & Walker, 1997; Callaway et al., 2002; Kikvidze et al., 2011; Pugnaire & Luque, 2001). However, others do not (see Maestre, Valladares, & Reynolds, 2006), where some studies demonstrate a null relationship between plant interactions on a stress gradient (Armas & Pugnaire, 2005; Casper, 1996; Tielbörger & Kadmon, 2000) or found that the competition is consistently intense across the stress gradient (e.g., Maestre & Cortina, 2004). Further, several studies also suggested that there is a humped shape pattern of plant–plant interactions along environmental stresses gradients (Forey, Touzard, & Michalet, 2009; Maalouf, Le Bagousse‐Pinguet, Marchand, Touzard, & Michalet, 2012; Smit, Vandenberghe, den Ouden, & Müller‐Schärer, 2007). This inconsistency in the findings of previous studies that conducted to examine the SGH could be due to shifts from negative to positive interactions influenced largely by the characteristics and ecological strategies of coexisting species (e.g., competitors versus stress tolerators—see Liancourt, Callaway, & Michalet, 2005; Maestre, Callaway, Valladares, & Lortie, 2009). For example, in a meta‐analysis, Gómez‐Aparicio (2009) found that the life form of the interacting species effect to a large degree the interactions outcome. Herbaceous plants, for instance, had negative effects on neighbors, particularly other herbaceous species, while shrubs had positive effects (Gómez‐Aparicio, 2009). Alternately, facilitation could break down, or competition could increase under extreme resource limitation in highly stressful environments (e.g., Michalet, Le Bagousse‐Pinguet, Maalouf, & Lortie, 2014).

Arid and semi‐arid lands are unproductive and stressful for the growth of plants. Nurse plants play an important role in ameliorating the environmental conditions and creating more benign microhabitats for the understory species in arid habitats (Abdallah & Chaieb, 2012; Anthelme & Michalet, 2009; Cortina et al., 2011). Positive interactions, or the nurse plant syndrome, tend to increase with increasing aridity (Flores & Jurado, 2003). The existence of nurse trees in arid environments creates a complex network of direct and indirect interactions between the nurse plant and the understory species around the nurse plant (see Cuesta, Villar‐Salvador, Puértolas, Rey Benayas, & Michalet, 2010; Michalet, Brooker, Lortie, Maalouf, & Pugnaire, 2015; Schöb, Armas, & Pugnaire, 2013). These interactions could occur between guilds (between the nurse tree and the understory species) or could occur within guilds (between the understory species; Weedon & Facelli, 2008). The extreme environment around the canopies of nurse plants in arid environments presents heterogeneity at the microhabitat scale (Gomez‐Aparicio et al. 2005). In this situation, interactions between species in communities under the nurse tree canopies may shift from facilitation at the stressful edge of the canopy to competition at the most benign center of the canopy. Stress gradients controlling biotic interactions in the small microhabitats under the canopy of nurse plants in arid environments may be fundamentally important in determining community composition and species distributions. As the nature of interactions between plants potentially changes across remarkably short environmental gradients, detecting competition or facilitation in these habitats may be highly dependent on where these communities are sampled. This effect may help to explain the inconsistent results of studies testing the stress gradient hypothesis.

The predictions of the net outcome of plant–plant interactions are usually based studies of pairwise species interactions (see Gómez‐Aparicio et al., 2004; He, Bertness, & Altieri, 2013; Maestre & Cortina, 2005). However, in natural communities, species interactions are not pairwise. Rather, species interact through complex multiple species interactions (i.e., neighborhood competition—see Keddy, 2001). Negative interactions between beneficiary plants under nurse plants have been observed in only a few studies (Schöb et al., 2013), and experimental evidence of competition between beneficiary plants is rare (e.g., Aguiar & Sala, 1994; Michalet et al., 2015). No studies have examined the potential for small‐scale stress gradients to allow plant–plant interactions to structure small plant communities in different microhabitats in extremely stressful landscapes.

We examined facilitation and competition under nurse trees in western Saudi Arabia. High daily temperatures, low soil moisture, and nutrient‐impoverished soil make much of this region extremely stressful and unproductive. However, between‐guild facilitation that occurs between the nurse tree (*Acacia gerrardii*) and understory vegetation creates productive microhabitats for understory vegetation. In a related study, we demonstrated that the under canopy microhabitats have high soil nutrients and water availability, and low light intensity and UV radiation relative to the surrounding areas (Al Namazi and Bonser, unpublished). In environments with *A. gerrardii* nurse trees, there are microhabitats differing in abiotic stress: relatively low‐stress habitats under canopies and relatively high‐stress habitats at the edge of canopies. We emphasize that these environments are quite stressful relative to mesic or temperate ecosystems. The distribution of herbaceous species in these arid environments tends to be habitat dependent. Some species are present at relatively high frequency under the canopy while others are present at relatively high frequency in the more stressful edge, and some even occur primarily in the open habitats (Al Namazi & Bonser, unpublished). These observations suggest that the distribution of species under the canopy of nurse plants could be controlled by increasing within‐guild competitive interactions on a gradient of decreasing abiotic stress toward the center of the canopy. Intriguingly, competition could play a major role in structuring communities in these extremely stressful habitats.

We conducted a neighbor removal experiment in the low‐stress canopy center microhabitats and high‐stress canopy edge microhabitats produced by nurse trees on four common perennial herbaceous species with different distributions under the canopies. We tested the following predictions: (1) interactions between understory species will shift from competition to facilitation in habitats of increasing stress from the center to the edge of *A. gerrardii* canopies, and (2) under canopies, species distributions will be limited by within‐guild competitive interactions at the center of the canopy, and facilitation at the edge of the canopy.

## 2 Materials and Methods

### 2.1 Study site

The study was conducted in Sederah Natural reserve in the National Wildlife Research Centre (NWRC), located on the arid Najd plains of western Saudi Arabia, about 45 km southern east of Taif Governorate in southwestern Saudi Arabia (21°14′55.6″N, 40°43′44.8″E). This reserve was declared as a nature‐reserved scientific center of 4 km^2^ fenced since 1986 by NWRC. Then, the area of the reserve was extended to comprise 19 km^2^ adjacent to the NWRC and fenced since 1992. The fence was established around this reserve to keep domestic livestock out, allowing the vegetation inside the protected area to recover from overgrazing. We conducted the study in the spring from March to June 2013. The annual average of rainfall in the reserve is about 85 mm. The annual average of the maximum and minimum air temperature is 37 and 15.7°C. This region experienced a drought during the winter months (several months before the experiment). However, during the experiment period (March, April, and May), the reserve received rainfall of 12.5, 21.1, and 11 mm in each month, respectively, and plant growth was sustained throughout the spring season despite the short rainy period.

### 2.2 Microclimate data

Soil temperature was 37°C under the canopy compared to 47°C immediately outside the canopy during the hours around midday. Mean photosynthetically active radiation (PAR) was 1930 ± 30 μmol/m^2^ s^−1^ outside the canopy (and 1700 μmol/m^2^ s^−1^ at the canopy edge) compared with 211 ± 14 μmol/m^2^ s^−1^ under the canopy (Al‐Namazi and Bonser, in review). We also sampled the soil from canopy center and edge microhabitats. Two soil samples from the canopy center and edge microhabitats under each of the ten trees were collected from the upper 10 cm of soil and combined. Soil samples were air‐dried, homogenized, and sieved. Chemical composition of the soil was analysed by the department of soil science at King Saud University, Riyadh, Saudi Arabia.

### 2.3 Study species

Trees in the genus *Acacia* are diverse and important in arid and semi‐arid ecosystems of the world (Ross 1981). Acacia trees contribute to increasing the productivity and diversity of understory plants that grow under their canopies (Abdallah & Chaieb, 2010, 2012; Belsky 1994; Ludwig et al. 2003). *Acacia gerrardii* (Benth.) is one of the most common trees in the arid and semi‐arid environments in Saudi Arabia.

We measured the mean abundance (the number of individuals per species in 1‐m^2^ quadrates) for each species in the understory, and the community density (the total number of individuals in 1‐m^2^ quadrates) under *A. gerrardii* canopies. Abundance and density were measured at the canopy center and edge microhabitats under each of the ten *A. gerrardii* canopies.

**Figure 1:**
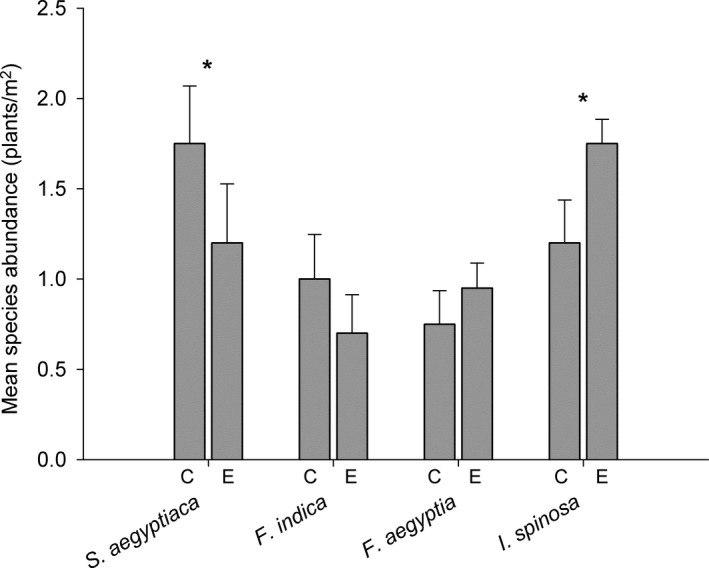
Mean (±*SE*) abundance of the four target species in the canopy center (C) and edge (E) microhabitats. * indicates instances where abundances for a given species were significantly different across habitats

Four short‐lived perennial herbaceous species commonly found under *A. gerrardii* nurse trees were selected for this study: *Salvia aegyptiaca* L. (Lamiaceae), *Fagonia indica* Burm. F. (Zygophyllaceae), *Farsetia aegyptia* Turra (Brassicaceae), and *Indigofera spinosa* Forsk (Fabaceae). Individuals of each species are found at both the center of the canopy and at the canopy edge, but these species are distributed differently under the nurse plant canopies (see Figure 1). *S. aegyptiaca* is an under canopy specialist (it is the dominant species under the canopy), *F. indica* is moderate canopy specialist (found more frequently under the canopy than at the canopy edge), *F. aegeptia* is a moderate edge specialist (it is found more frequently at the edge of the canopy than at the center of the canopy), and *I. spinosa* is an edge specialist (it is the dominant species at the edge of the canopy). *I. spinosa* is also a nitrogen fixer (Sprent & Sprent, 1990). Species were either absent outside the canopy or were present in small numbers.

### 2.4 Experimental design

We conducted a neighbor removal experiment to test our predictions on interactions between species under nurse plant canopies. Young plants of the four target species that already established under *A. gerrardii* trees were selected as target plants. We recorded the size (plant height and canopy width) of the young plants at the start of the experiment. Target plants were selected from two microhabitats: close to the center of the canopy and near the edge of the canopy. Central canopy plants were located within 1 m of the tree trunk. Edge plants were located from 50 cm inside the canopy to 50 cm outside the canopy. Target plants were identified under 10 *A. gerrardii* trees. The canopy diameter of the *A. gerrardii* nurse trees ranged from 6 to 14 m. Target plants from each microhabitat were randomly assigned to one of two competition treatments: neighbors removed and neighbors left intact. Under the neighbor removal treatment, target plants were centered in a circular plot 50 cm in diameter. All neighbor plants within the plot were removed to ground level at the start of the experiment at the first week of April 2013. Under the neighbors left intact treatment, target seedlings were grown under natural neighboring vegetation (mostly other individuals of the four species selected for the experiment). We did not remove underground plant material in the neighbor removal treatment. The roots neighboring plants would be difficult to excavate while leaving the roots of the target plant intact. This is a common approach for neighbor removal experiments (see Aarssen & Epp, 1990). Further, the remaining root tissue would not likely significantly decompose and increase soil nutrients over the time frame of the experiment (see Lamb, Kembel, & Cahill, 2009). Competition treatments were replicated 10 times at each microhabitat for each of the four experimental species. Target plants were harvested, and the experiment was completed at the end of May 2013. Our experiment ran over the primary growing season for the year in these habitats, as most species have only limited growth in the summer season, and individuals either remain dormant or die during the harsh dry season after the short rainy period.

### 2.5 Data collection

We examined the impact of neighbors on the growth of target species in the edge and center microhabitats. At the beginning of the experiment, 20 nonexperimental individuals of each species of the four target species in various sizes were randomly collected to estimate the dry mass of plants at the beginning of the experiment. The dry mass, height, and diameters of these individuals were used in the linear regression model in order to obtain a regression model for estimating the dry mass depending on the heights and diameters of the experimental target seedlings. This regression was used to estimate the initial dry mass of the seedlings of target species based on their heights and diameters at the beginning of the experiment. At the end of the experiment, the aboveground material of target species was harvested to measure the final dry mass. To estimate the dry mass of the seedlings and experimental target plants, aboveground plant material for each plant was placed individually in paper bags and dried in a drying oven at 60°C for 72 hr.

### 2.6 Data analysis

To compare competition and facilitation effects across the stress gradient under the canopy, we used the index of relative neighbor effect (RNE, Markham & Chanway, 1996), using aboveground biomass as the response units. This index is calculated as follows:

$$\text{RNE}=(B_{T-N}-B_{T+N})/X$$

where *B*_T−N_ is the biomass of aboveground part of the target plant with neighbors removed, while *B*_T+N_ is the biomass of aboveground part of the target plant with neighbors left intact, and *X* is the max of (*B*_T−N_, *B*_T+N_). This index ranges from −1 to 1, the negative values indicate facilitation between neighbors, and positive values indicate competition.

The effect of neighbors on the performance of the four target species was estimated by examining differences in growth across competition treatments for each species in different microhabitats. As the neighbor removal experiment is conducted on already established plants, the initial biomass of species individual could vary among such species individuals. To control for this issue, we estimated the change in growth using a growth index by measuring the accumulation of biomass of plants controlling for size at the beginning of the experiment and the final biomass at the end of experiment using the following formula:

$$\text{Growth Index}=\left(\text{Dry}\;{\text{Mass}}_{t2}-\text{Dry}\;{\text{Mass}}_{t1}\right)/\text{Dry}\;{\text{Mass}}_{t1}$$

where Dry Mass_*t*1_ is the dry mass at the beginning of the experiment, and Dry Mass_*t*2_ is the dry mass at the end of the experiment. We used mixed‐model analysis of variance to examine variation in target plant growth due to species, microhabitat, and competition treatment. The species effect (and interaction terms including species) was included as a random effect while microhabitat and competition were fixed effects. Significant differences in mean growth values were assessed using Tukey's HSD. Significant differences between target species' abundance and soil characteristics were assessed using two‐sample general linear models. Data were analysed with SPSS 16.0.

## 3 Results

Plants in the canopy edge microhabitat experience significantly higher abiotic stress than plants in the canopy center microhabitat. The edge microhabitat habitat had higher daytime temperatures and light intensity (see Methods) than the canopy center microhabitat. Further, the soil at the edge microhabitat had significantly lower magnesium, potassium, and sulfate than the canopy center microhabitat (Table 1). Community density was higher at the canopy center (10.4 ± 1.01 individuals per m^2^) than at the canopy edge (8.3 ± 0.08 individuals per m^2^).

**Table 1:** The mean (±*SE*) for soil features through the two microhabitats: under canopy (Canopy) and canopy edge (Edge). Soil features included are nitrogen (N), phosphorus (P), organic matter (OM), manganese (Mn), pH, clay content (Cl), sulfate (SO^−^_4_), calcium (Ca^+^_2_), magnesium (Mg^+^_2_), sodium (Na^+^), and potassium (K^+^). The *F* and *p* values report the significance of the differences between soil features in canopy and edge microhabitats

	N	P	OM	Fe	Mn	pH	Cl	SO^−^_4_	Ca^+^_2_	Mg^+^_2_	Na^+^	K^+^
	(ppm)	(ppm)	(%)	(ppm)	(ppm)		(mg/100 g)					
Canopy	1086 ± 160	513 ± 49	0.833 ± 0.24	8807 ± 504	135 ± 7.2	7.72 ± 0.13	0.64 ± 0.16	1.25 ± 0.36	1.62 ± 0.25	0.46 ± 0.17	0.38 ± 0.09	0.37 ± 0.036
Edge	871 ± 152	533 ± 52	0.42 ± 0.08	9460 ± 534	142 ± 6.9	8.1 ± 0.09	0.50 ± 0.1	0.38 ± 0.11	0.9 ± 0.18	0.15 ± 0.01	0.20 ± 0.016	0.24 ± 0.032
*F*	0.95	0.079	2.75	0.791	0.42	5.127	0.5	5.3	5.417	4.30	5.16	7.38
*p*	.34	.78	.113	.385	.525	.035	.487	.032	.031	.051	.034	.013

We found a significant effect of neighbor removal on the growth of target plants. However, the effect of neighbor removal was highly species and habitat dependent (i.e., a significant three‐way interaction between these effects, Table 2). In addition, we found significant species and habitat effects on growth, and interactions between the main effects were also significant, with the exception of species × habitat and species × neighbor removal (Table 2).

**Table 2:** Mixed‐model analysis of variance results on the impact of species, canopy position, and neighbor removal on plant growth. Species (and interaction terms including species) were included as random effects

Source of variation	Sum of squares	*df*	MS	*p*
Species (S)	38.89	3	13.3	<.0001
Canopy position (CP)	3.80	1	3.8	<.0001
Neighbor removal (NR)	1.0	1	0.96	.046
S × CP	1.89	3	0.63	.052
S × NR	0.73	3	0.24	.387
CP × NR	5.67	1	5.67	<.0001
S × CP × NR	3.23	3	1.08	.005
Error	77.071	156	0.494	

**Figure 2:**
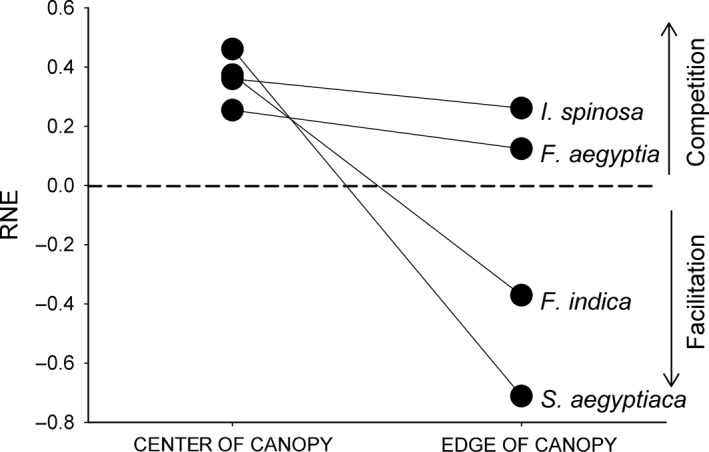
The index of relative neighbor effect (RNE) for the four target species (*Salvia aegyptiaca*,* Fagonia indica*,* Farsetia aegyptia*, and *Indigofera spinosa*), through two microhabitats: under the center of the canopy and at the edge of canopy. Values above the dashed line indicate competitive interactions, and values below the dashed line indicate facilitation interactions

The index of relative neighbor effect (RNE) of target species was variable through the two microhabitats (Figure 2). Interactions between plants were generally competitive under the canopy, but shifted to facilitation at the edge of the canopy. The relative neighbor effect (RNE) values of the four species were variable among species in both microhabitats. All four species have positive RNE values at the center of the canopy, and these values decrease at the canopy edge (Figure 2). The RNE values of two of the four species (*F. indica* and *S. aegyptiaca*) decreased dramatically from positive (0.37 and 0.46, respectively) to negative values (−0.37 and −0.71, respectively). This indicates that the canopy‐dominant and moderate canopy species (*S. aegyptiaca* and *F. indica*) experienced competition from neighbors in the less stressful microhabitat and facilitation in the more stressful microhabitat. In contrast, the RNE values of the edge specialist and moderate edge species (*F. aegyptia* and *I. spinosa*) decreased, but RNE values were positive in both microhabitats. This indicates that the individuals of *F. aegyptia* and *I. spinosa* experienced competition across the stress gradient.

**Figure 3:**
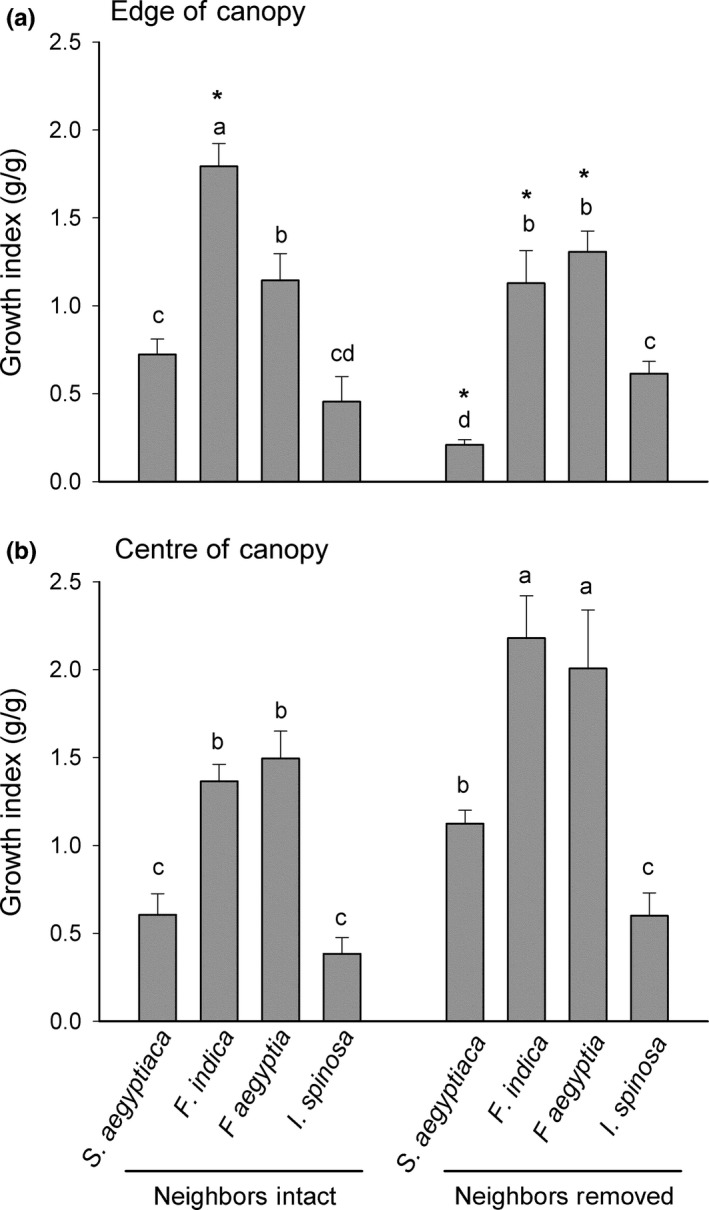
Mean (±*SE*) of growth index of the four target species presented from canopy to edge specialist (*Salvia aegyptiaca*,* Fagonia indica*,* Farsetia aegyptia*, and *Indigofera spinosa*), under two treatments (neighbors removed and neighbors left intact) through two microhabitats: (a) at the edge of the canopy and (b) at the center of the canopy. Significantly different species and treatment means within edge or center microhabitats are indicated with different letters. * indicates a significantly different mean for a given treatment and species combination across canopy center and edge microhabitats

The significance of the neighbor effects (e.g., the shift from competition to facilitation) is derived from the ANOVA (Table 2; Figure 3). Differences in growth in different canopy positions and under neighbor removal treatments explain changes in competition and facilitation experienced by the different experimental species (Table 2). For example, growth in *F. indica* and *S. aegyptiaca* (the two canopy specialist species) was significantly higher in the neighbor removal treatment at the center of the canopy. At the edge of the canopy, the growth of these two species was significantly higher in the presence of neighbors than that in the absence of neighbors (Figure 3). In contrast, the growth of *F. aegyptia* and *I. spinosa* (the two canopy edge species) remains higher in the absence of neighbors in the two microhabitats although the growth of these two species was not consistent between two microhabitats. The growth of *F. aegyptia* decreased between center and edge habitats in both neighbor removal treatments, and was significantly greater at the center of the canopy than at the canopy edge in neighbor removal treatments. The growth of *I. spinosa* increased slightly but not significantly between center and edge habitats in the neighbors intact treatment, but not in the neighbor removal treatment (Figure 3). While *F. indica* and *S. aegyptiaca* were more abundant in the canopy center microhabitat, the growth of each target species was reduced by neighbors in this microhabitat, and this effect was relatively consistent across species (Figures 2, 3).

## 4 Discussion

We found complex interactions among beneficiary species that grow under a nurse plant (*Acacia gerrardii*). These within‐guild interactions shift from competition to facilitation with increasing stress from the center to the edge of nurse tree canopies. Although our finding was observed over very small stress gradients under nurse trees, this observation supports the stress gradient hypothesis frequently observed across broad environmental gradients (Bertness & Callaway, 1994). However, the stress gradients determining the nature of species interactions occur at remarkably fine scales within these stressful arid environments, rather than at landscapes scales where the stress gradient hypothesis is typically tested (Sthultz, Gehring, & Whitham, 2007; Tewksbury & Lloyd, 2001).

In arid regions of the Arabian peninsula, *Acacia* trees represent “islands of fertility” with higher soil moisture and nutrient contents under the canopies than the surrounding harsh environments (Abdallah & Chaieb, 2010; Robinson, 2004). We found that the stress gradient generated by *A. gerrardii* canopies plays a significant role in structuring the understory herbaceous communities. On average, the intensity of competition experienced by the four target species under canopy (the less stressful microhabitat) was greater than that of their counterparts growing at the edge of the canopy (the more stressful microhabitat) of *A. gerrardii*. These results are consistent with the predictions strategy theory that the intensity of competition increases with decreasing the stress of habitat (Grime, 1977). However, decreasing competitive interactions with increasing stress may not simply be due to less intense competition between plants in more stressful environments. Rather, our results suggest that the strong positive effects of neighbors in these stressful environments can outweigh the negative effects of ongoing resource competition. For example, the net outcome of the interaction between two of the four species (*F. indica* and *S. aegyptiaca*) and their neighbors shifted from competition in the less stressful microhabitat (under canopy) to facilitation in the stressful environmental microhabitat (at the edge of canopy). In the less stressful microhabitat, these species performed better in the absence of neighbors, while in the more stressful microhabitat at the edge of canopy, plants grew performed better in the presence of neighbors. The other two species (*F. aegyptia* and *I. spinosa*) always performed better in the absence of neighbors, but this difference was only significant for *F. aegyptia* in the less stressful microhabitat. Overall, there is a shift in how biotic interactions between herbaceous species structure these communities from competition at the center of the canopy, to increasing facilitation at the edge of the canopy.

Coexisting species do not respond the same way to biotic interactions under the same environmental conditions. Competition limits the growth of most species at the center of the canopy except *I. spinosa*, a species with low growth across neighbor removal treatments in both the edge and canopy center habitats (see below). The higher abundance of *F. indica* in the canopy center microhabitat is perhaps due to a high competitive ability, and fast growth even in the presence of neighbors at the center of the canopy. However, *F. aegyptia* also grew well in the presence of neighbors in the center of the canopy, even though it is found more frequently in the more stressful microhabitat. Interestingly, *S. aegyptiaca* (the under canopy specialist species) grew relatively severely, even in the under canopy microhabitat, and its growth reduction in the presence of neighbors was similar to that of the other species. Perhaps competitive superiority in these habitats is achieved by a capacity to reproduce quickly and efficiently under competition (e.g., Bonser, 2013; Tracey & Aarssen 2011) particularly as the growing seasons in these regions tend to be quite short. In the year we conducted our experiment, many plants had not started reproducing by the onset of the dry season (the wet season was short compared to other years, and reproduction may have been lower than average years). Alternately, persistence across the dry seasons could also increase species abundance in these habitats. Future studies will need to investigate differences in reproduction under competition, and in the capacity for understory herbaceous species to survive the long dry seasons in these regions to better understand the dominance of some species in the low‐stress microhabitats.

At the canopy edge, abiotic stress increases its importance in controlling species abundance, but a primary impact of abiotic stress appears to be in modifying biotic interactions. For example, species more abundant in the canopy center microhabitat grew very poorly in the absence of neighboring plants in the edge microhabitat. These species appear to have low stress tolerance, and facilitation from neighboring herbaceous vegetation can promote their persistence in the higher stress canopy edge microhabitat. In contrast, the two edge specialist species (*I. spinosa* and *F. aegyptia*) did not rely on facilitation in the more stressful microhabitat. In particular, growth of *I. spinosa* did not differ across neighbor removal treatments or canopy position, which is consistent with a stress‐tolerant strategy (Grime, 1977). A stress‐tolerant species such as *I. spinosa* will tend to grow slowly compared to other, less stress‐tolerant species across competition treatments. Slow growth limits the success of these species at the center of the canopy where they are likely competitively displaced by fast growing and competitively superior species. The stress‐tolerant plants do not benefit from facilitation in the stressful edge sites, but the non‐stress‐tolerant (competitor) species do benefit greatly from facilitation promoted by stress‐tolerant plants at the edge of the canopy. In addition, *I. spinosa* is a nitrogen fixer—a trait characteristic of facilitator species (Bonanomi et al., 2011). These understory communities rely on facilitation from nurse trees to create the environmental conditions amenable for the assembly of these relatively productive herbaceous communities. While the overall biotic interactions shift from competition to facilitation from the low‐stress to high‐stress habitats established by these nurse plant canopies, the nature of interacting species and their life‐history characteristics play a vital role in the interactions between species (Liancourt et al., 2005; Maestre et al., 2009).

Our experiment was conducted over a single growing season. Recent research suggests that the interactions between nurse plants and understory herbaceous plants suggest that the short‐term and long‐term effects can be quite different, and long‐term effects are perhaps more important (Noumi, Chaieb, Le Bagousse‐Pinguet, & Michalet, 2016). The interactions between understory species may vary over time, and growing seasons where competition in the canopy center habitat is relatively low (and facilitation is high) could promote the persistence of stress‐tolerant species at the canopy center. The dominance of more stress‐tolerant plants at the canopy edge would also likely result in a lower competitive effect of neighbors in the more stressful microhabitat. A lower competitive effect would contribute to lower competition intensity under high stress (Liancourt et al., 2005). However, species distributions under nurse tree canopies are likely the outcome of long‐term interactions and species distributions are broadly consistent with the hypothesis that interactions between understory species shift from competition to facilitation with increasing stress under nurse tree canopies.

The presence of a strong facilitator or nurse species in an extremely stressful environment establishes environmental conditions that promote competitive interactions and limit the impact and importance of facilitation, even at the most stressful ends of broad geographic stress gradients. The modest and sometimes equivocal support of the stress gradient hypothesis is potentially due to either a breakdown of facilitation in the highest stress environments or a switch from facilitation to competition when resources are most limited (Michalet et al., 2014). Our results suggest that facilitation is central to community structure in high‐stress environments. Further, we did not find evidence that competition will increase in importance as resources become critically limited—although the understory habitats in our study may not have crossed a threshold for resource limitation required to induce competition. Alternately, the lack of general support for the SGH could be due to neglecting the variation in life‐history and ecological strategies of interacting species and the nature of the stress (Maestre et al., 2006, 2009). We demonstrate that detecting competition and facilitation interactions are both possible (and perhaps likely) in arid or stressful environments, even under environmental conditions necessitating facilitation to establish plant communities. Details of the ecological strategies of coexisting species and the strength of interactions between nurse plants and beneficiary species are important in understanding the conditions where competition will emerge and control community composition in stressful environments.

In conclusion, our results show that the distribution of herbaceous species under the canopy of nurse plant in the arid environment is controlled by a complex interplay between the abiotic stresses established by the nurse trees, and the interactions between coexisting species. The nature of interacting species (i.e., competitive ability and stress tolerance) likely controls the outcome of these interactions. In the low‐stress microhabitat, the dominant species should have high competitive ability. However, facilitation by stress‐tolerant species and the capacity to tolerate environmental stress control the persistence of species in the more stressful canopy edge habitats. Overall, competition and facilitation play key roles in the distribution of species and the assembly of communities under nurse trees in these extremely stressful habitats. Our results are important in understanding how competition and facilitation control community assembly on stress gradients.

## Conflict of interest

None declared.
